# Outcomes of endoscopic third ventriculostomy (ETV) and ventriculoperitoneal shunt (VPS) in the treatment of paediatric hydrocephalus: Systematic review and meta-analysis

**DOI:** 10.1007/s00381-023-06225-3

**Published:** 2023-11-27

**Authors:** Katarzyna Julia Minta, Siddarth Kannan, Chandrasekaran Kaliaperumal

**Affiliations:** 1 University of Aberdeen School of Medicine Medical Sciences and Nutrition, Polwarth Building, Foresterhill Rd, Aberdeen, AB25 2ZD UK; 2 School of Medicine, University of Central Lancashire, Preston, UK; 3 Department of Clinical Neuroscience, Royal Infirmary of Edinburgh, Edinburgh, UK

**Keywords:** Hydrocephalus, Endoscopic third ventriculostomy, Ventriculoperitoneal shunt, Management

## Abstract

**Purpose:**

To evaluate the outcomes of endoscopic third ventriculostomy (ETV) and ventriculoperitoneal shunt (VPS) in the treatment of paediatric hydrocephalus.

**Methods:**

We searched PubMed, MEDLINE, and Cochrane Central Register of Controlled Trials databases for articles published from 2000 to May 2023 (last search date May 6, 2023). Keywords searched included “endoscopic third ventriculostomy”, “ventriculoperitoneal shunting”, “paediatric population”, and “outcomes”. Using random-effects models, we compared success rates and complications of ETV and VPS. The primary outcome was ETV vs.VPS success rates, and the secondary outcome was post-treatment complications. Included studies reported on treatment success and complication rates.

**Results:**

Out of 126 articles, 8 RCTs and 1 prospective study were included. Six studies reported primary outcome data (806 patients identified: 464 in ETV group, 342 in VPS group). Combined success rates were 81.8% (*n* = 283/346) for ETV and 86.7% (*n* = 182/210) for VPS (median follow-up 41 months). There was no difference in success rates between ETV and VPS groups (risk ratio 0.84, 95% confidence interval 0.80–0.90, *I*^2^ = 0%, *p* = 0.93). Combined complication rates were 4.6% (*n* = 16/346) in the ETV group and 27.1% (*n* = 57/210) in the VPS group. ETV had a lower rate of postoperative complications (risk ratio 0.76, 95% confidence interval 0.42–1.38, *I*^2^ = 53%, *p* = 0.04).

**Conclusions:**

Both ETV and VPS are viable surgical options for the management of paediatric hydrocephalus with similar success rates when used as first-line treatment. However, our study concluded that VPS results in a higher complication rate.

**Registration:**

This systematic review and meta-analysis was formally registered in the PROSPERO International database under the registration number CRD42023452907 on the 29^th^ of August 2023.

**Supplementary Information:**

The online version contains supplementary material available at 10.1007/s00381-023-06225-3.

## Introduction

Hydrocephalus is a neurological condition characterised by the accumulation of cerebrospinal fluid (CSF) within the ventricular system of the brain. It is a prevalent disorder among paediatric patients, often requiring surgical intervention for the management of symptoms and prevention of long-term complications. It commonly develops from a congenital brain malformation or after sustaining a neurological insult for instance bleeding or infection [1]. In the case of hydrocephalus occurring after a complication for instance haemorrhage, neoplasm, or infection, then it is referred to as secondary hydrocephalus. Unfortunately, hydrocephalus is a major cause of mortality and morbidity in a paediatric population accounting for 100,000–200,000 new cases each year in sub-Saharan Africa [2, 3].

Two commonly employed surgical procedures for the treatment of paediatric hydrocephalus are endoscopic third ventriculostomy (ETV) and ventriculoperitoneal shunt (VPS). Shunts are mechanical devices which are associated with numerous complications for instance obstruction, failure, or infection. Unfortunately, there is an increased risk of mortality related to delayed recognition of shunt malfunction [4]. This study evaluated two key outcomes that are success rate and postoperative complications. The first metric of “success rate” which is defined as the percentage of patient cases in which a shunt used to treat hydrocephalus remains functional and does not require revision or replacement at 1-year time due to complications such as shunt malfunction, infection, or blockage. Further, another metric used in this study was complication rates post-surgery which refers to the number of patients who experience adverse events or issues following the surgical procedure. The postoperative complications delineated in this study and pooled in the final analysis included infections, seizures, CSF leak, and subdural haematomas [4].

ETV involves creating an artificial communication between the floor of the third ventricle and the subarachnoid space, allowing for the diversion of CSF and the restoration of normal intracranial pressure. This approach mitigates the risk of infection associated with hardware or shunt malfunction particularly in developing countries. Conversely, VPS involves the placement of a catheter into the ventricles, which then drains the excess CSF into the peritoneal cavity. Both procedures aim to alleviate the symptoms associated with hydrocephalus and improve the patient’s quality of life [5].

The choice between ETV and VPS is influenced by several factors, including the aetiology of hydrocephalus, patient age, anatomical considerations, and surgeon expertise [6]. Both procedures have shown promising results in individual studies, yet considerable debate persists regarding their comparative effectiveness and safety in paediatric patients [7].

### Objectives

The primary objective of this study was to evaluate the outcomes of endoscopic third ventriculostomy and ventriculoperitoneal shunt as a treatment of choice for paediatric population hydrocephalus. The secondary objective of this systematic review and meta-analysis was to characterise the scope and the quality of the current literature on hydrocephalus in paediatric population. Any other forms of surgery such as choroid plexus cauterization (CPC) was not included due to limited amount of literature.

## Methodology

### Electronic literature search

**Fig. 1:**
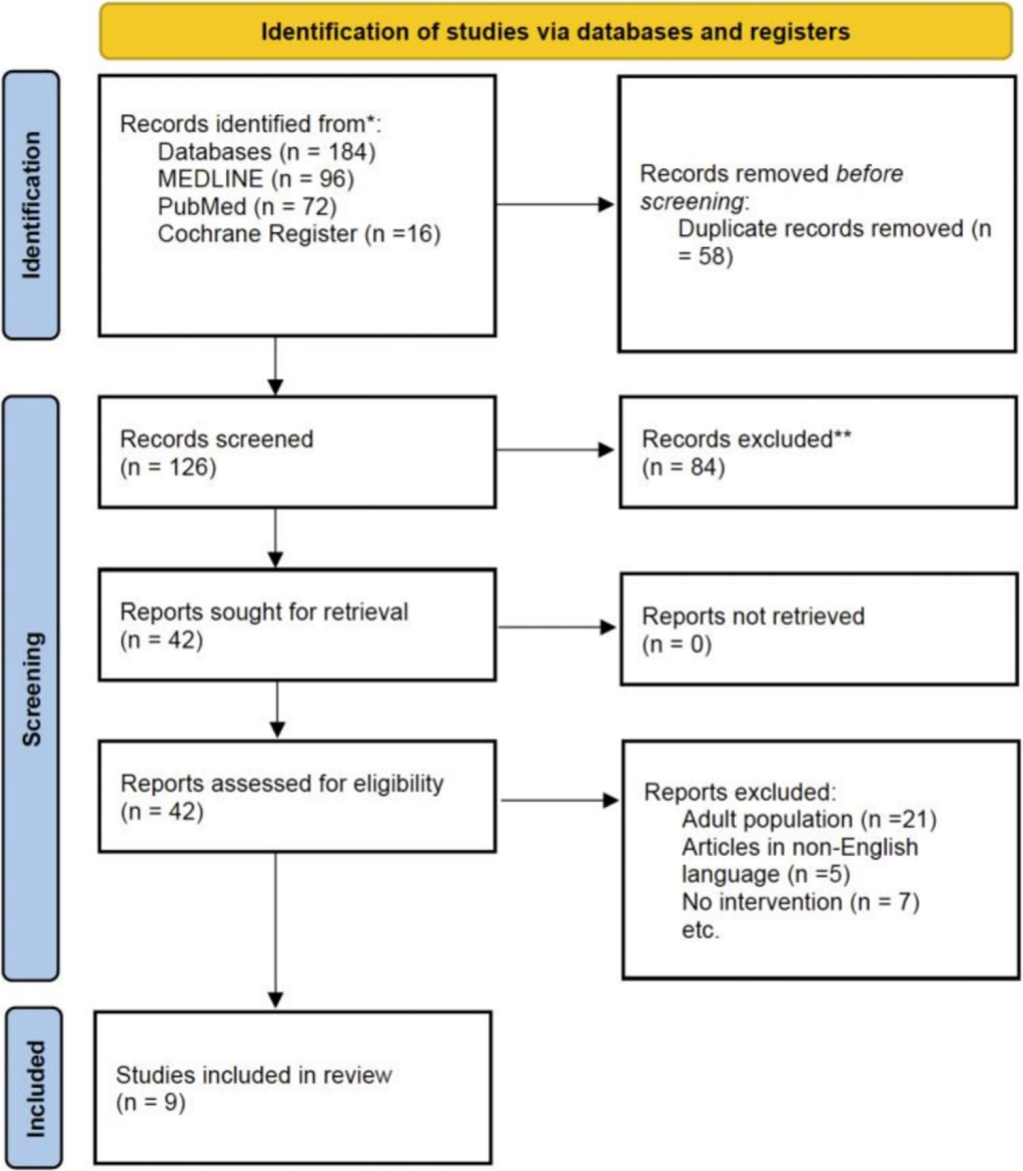
Preferred Reporting Items for Systematic Reviews and Meta-Analysis (PRISMA) [8]

This systematic review and meta-analysis has been conducted in line with the Preferred Reporting Items for Systematic Reviews and Meta-Analysis (PRISMA) guidelines as demonstrated in Fig. 1. This systematic review and meta-analysis have been registered prospectively in the international register PROSPERO under the registration number CRD42023452907.

#### Database and search strategy

PubMed, MEDLINE, and Cochrane Central Register of Controlled Trials databases were screened from April 3, 2023, until May 22, 2023. The search strategy was based on the utilisation of MeSH (Medical Subject Headings) term and each keyword search of each database was conducted based on the Boolean operators AND and OR. The searched keywords utilised in the article screening process included the following: “endoscopic third ventriculostomy”, “ventriculoperitoneal shunt”, “surgical management”, and “paediatric hydrocephalus”. The complete search protocol can be viewed in Appendix A. Thereafter, the data was uploaded to R programme for statistical analysis. We have reported our results based on descriptive statistics analysis and the forest plots obtained.

### Eligibility criteria

#### Inclusion criteria

Inclusion criteria were as follows: randomised controlled trials, studies involving patients who had undergone an endoscopic third ventriculostomy or ventriculoperitoneal shunt as an intervention, studies involving paediatric population, studies involving human participants and including both genders, available full-text articles published in English, patients who have hydrocephalus, studies found on the Ovid MEDLINE, Cochrane Register of Clinical Trials, Embase, and Google Scholar databases, and the timeline of RCTs was set between 2000 until present. The minimum sample size of the population was set to 10 participants for each RCT.

#### Exclusion criteria

Non-original articles such as editorials, letters to the editor, and conference abstracts were excluded, as well as articles in languages other than English. Articles reporting findings of the RCTs from adult population were excluded as well as articles published before 2000.

### Study selection

Two authors KJM and SK independently assessed the titles and abstracts and retrieved relevant articles to evaluate the relevance. Articles based on titles and abstracts of all extracted articles which met the inclusion criteria were selected for further review with all duplicate removed at this point. Thereafter, full articles were retrieved for all studies which met the inclusion criteria. Further, the same two authors independently assessed the full text for applicability. Any disagreements among the two authors were resolved by discussion and percent agreement and where necessary the senior supervisor would reach a final decision. The reasons for exclusion of the full text were noted. There was no blinding of reviewers towards the journal titles, institutions, or study authors.

### Data synthesis and quality assessment

Following the literature search and screening against the inclusion and exclusion criteria, 8 RCTs a 1 prospective study were included in the final analysis. The baseline characteristics of the population and outcomes measured are summarised in Table 1.

**Table 1:** Baseline characteristics and outcomes measured for all the studies included in the final analysis

First author	Year of publication	Study design	Total no of patients	Treatment modality	Mean age (months)	No of patients	Success rate (%)	Complication rate	Mean hospital stay (days)
Kulkarni [9]	2016	Randomised controlled trial	158	ETV	4.3 (1.8–7.7) - months	115	77/115 (66%)	7/115	5 (4–10)
				VPS	2.2 (0.6–5.3) - months	43	34/43 (79%)	0/43	5 (4–9)
Malheiros [3]	2010	Prospective study	17	ETV	37 months	10	8/10 (80%)	0/10	3.3 days
				VPS	36 months	7	5/7 (71.5%)	0/7	3.1 days
Kestle [10]	2003	Randomised controlled trial	393	ETV	84 days - median	194	128/194 (65%)	18/194	-
				VPS	110 days - median	199	159/199 (79%)	11/199	-
Kulkarni [1]	2018	Randomised controlled trial	78	ETV	5.6 (5.2) - age in months at first surgery, mean, SD	59	38 (64.4%)	3/59	7.7 (5.8)
				VPS	3.8 (5.6) - age in months at first surgery, mean, SD	19	15 (78.9%)	2/19	8.5 (8.8)
Kulkarni [11]	2017	Randomised controlled trial	100	ETV	3.1 (2.2 to 4.2) - months	51	33/51 (65%)	2/51	-
				VPS	3.1 (2.7 to 3.9) - months	49	37/49 (76%)	2/49	
Schiff [12]	2021	Randomised controlled trial	100	ETV	-	51	-	2/51	-
				VPS		49		2/49	
Haq [13]	2022	Randomised controlled trial	60	ETV	31.5 ± 6.31	35	25/35 (83.3%)	5/30	3.4 ± 6.45
				VPS	32.3 ± 5.46	25	20/25 (66.7%)	10/30	8.1 ± 1.35 days
Punchak [6]	2019	Randomised controlled trial	100	ETV	3.25 months	51	-	10/51	-
				VPS		49		10/49	
Lane [7]	2022	Randomised controlled trial	100	ETV	11–24 months	51	-	10/43	-
				VPS		49		38/57	

### Risk of bias assessment

The included studies were assessed using the Cochrane risk-of-bias tool for randomised trials (RoB 2) [14] by two independent researchers. In the case of disagreements, a third researcher provided input to reach a consensus. The risk of bias was assessed in seven domains and categorized as “low risk of bias”, “some concerns of bias”, or “high risk of bias” (Table 2).

**Table 2:** Types of hydrocephalus included in study

First author	Type of hydrocephalus
Kulkarni [1]	Triventricular hydrocephalus (TVH)
Malheiros [3]	Not mentioned
Kestle [10]	Obstructive
Kulkarni [9]	Triventricular hydrocephalus (TVH)
Kulkarni [11]	Postinfectious
Schiff [12]	Postinfectious
Haq [13]	Obstructive
Punchak [6]	Post infectious
Lane [7]	Post infectious

## Results

### Study selection

Two independent reviewers completed the study selection. The database search yielded 184 articles (PubMed, 72; MEDLINE, 96; and Cochrane Central Register of Controlled Trials databases, 16). After the removal of 58 duplicates, 126 articles remained. These were assessed against a predetermined exclusion criteria by title and abstract. Eighty-four were excluded due to inconsistent research objectives. Furthermore, the full text of the remaining 42 articles was attempted to be retrieved. The full text of 42 articles was assessed against the exclusion criteria. Nine articles were consistent with the inclusion criteria and so were included in this review. The same two independent reviewers that conducted the database search also carried out this process. The full search strategy is available as Appendix A.

### Population

Paediatric population patients with hydrocephalus.

### Intervention

Patients who have undergone endoscopic third ventriculostomy or ventriculoperitoneal shunt as an intervention.

### Comparison

To compare the effectiveness of endoscopic third ventriculostomy and ventriculoperitoneal shunt in the management of hydrocephalus in paediatric population.

### Outcome

The primary outcomes of interest were treatment success. The secondary outcomes measured were complication rates.

### Grouping analysis of studies

#### Baseline characteristics

The baseline characteristics of included studies are summarised in Table 1. Most common country of publication was Africa. The total number of patients was 1106 patients, with a median number of patients treated via ETV: 566 and VPS: 540.

### Risk of bias assessment

The assessment of bias was done via the Cochrane risk-of-bias tool assessment shown in Table 3 [14].

**Table 3:**
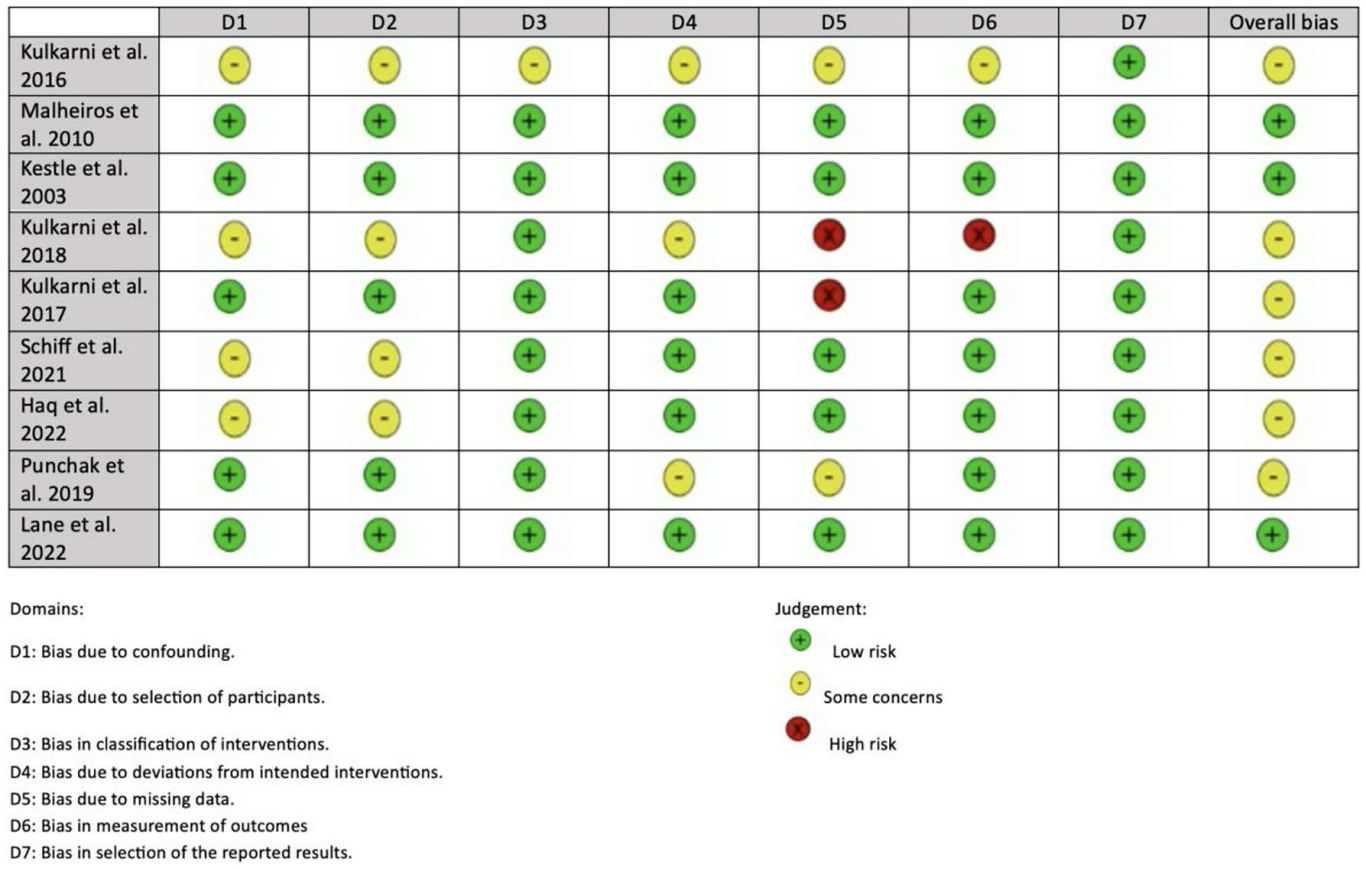
Summary of risk of bias assessment using revised Cochrane risk-of-bias tool for randomised trials (RoB 2)

### Statistical analysis

Data was extracted via a Microsoft Excel spreadsheet, then exported to R version 4.0 for analysis and figure generation. Baseline demographics were summarised using descriptive statistics. Symptom and patient counts were summarised using means and standard deviation (SD), and if not normally distributed, using a median and interquartile range (IQR [15]). For the meta-analysis, we used a random-effects model for pooled proportion analysis, and meta-analysis of diagnostic accuracy studies, in accordance with published guidelines [16.] We generated forest plots for studies that included proportion, pooled sensitivity, and specificity. Heterogeneity was assessed using the I2 characteristic. Publication bias was evaluated and presented as funnel plots. R statistics (Rstudio Version 4.0.1) was used to perform meta-analysis and create forest and funnel plots (ggplot, tidyverse, metafor, metaprop, mada, and meta packages).

#### Treatment success

**Fig. 2:**
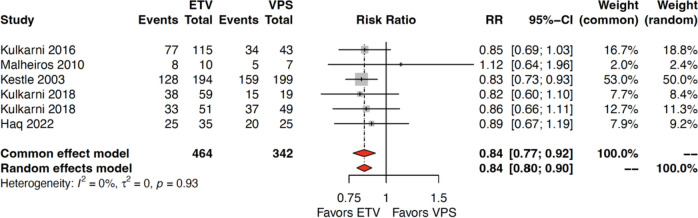
Forest plot depicting success rate outcome for ETV and VPS group of patients respectively

Six studies reported treatment success rates as a primary outcome (806 patients identified: 464 in ETV group, 342 in VPS group). Combined success rates were 81.8% (*n* = 283/346) in the ETV group and 86.7% (*n* = 182/210) in the VPS group (median follow-up 41 months). There was no difference in success rates between ETV and VPS groups (risk ratio 0.84, 95% confidence interval [0.80–0.90], *I*^2^ = 0%, *p* = 0.93) (Fig. 2).

#### Complication rates

**Fig. 3:**
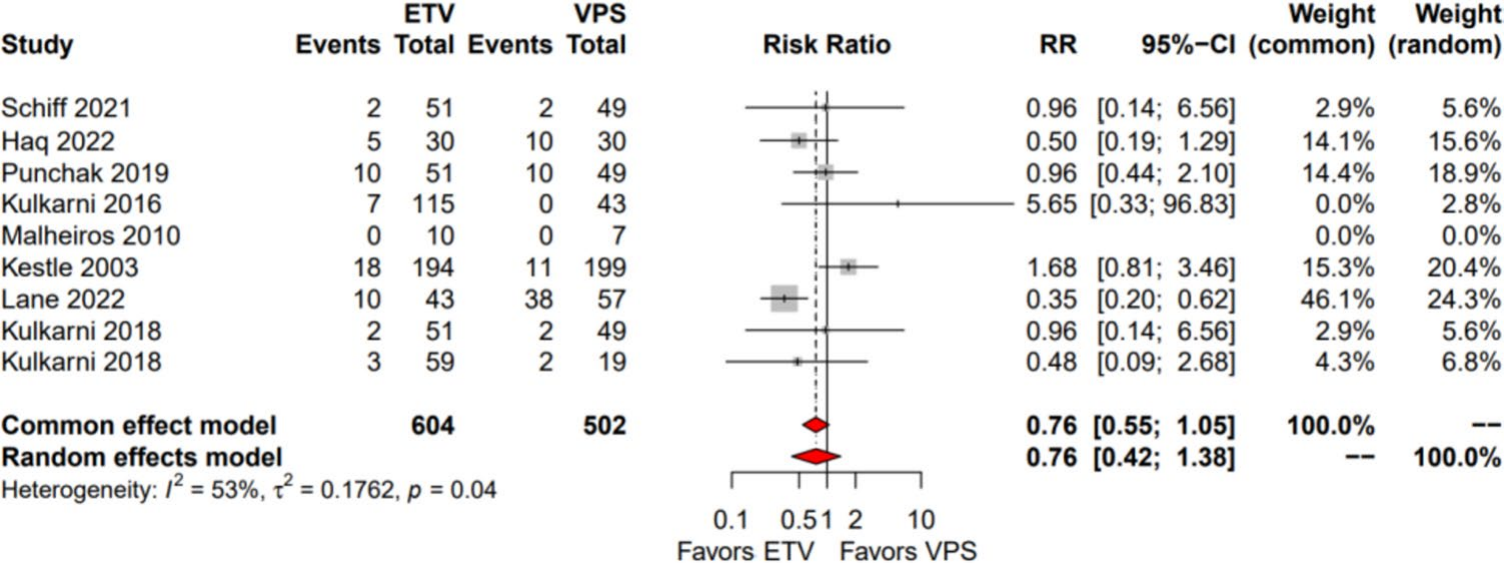
Forest plot depicting postoperative complication rate for ETV and VPS group of patients respectively

Nine studies reported postoperative complication rates as a primary outcome (1106 patients identified: 604 in ETV group, 502 in VPS group). Combined complication rates were 4.6% (*n* = 16/346) in the ETV group and 27.1% (*n* = 57/210) in the VPS group. ETV had a lower rate of postoperative complications (risk ratio 0.76, 95% confidence interval [0.42–1.38], *I*^2^ = 53%) (Fig. 3).

## Discussion

### Summary of findings and future implications

This systematic review and subsequent meta-analysis revealed comparable success rates between ETV and VPS. Nevertheless, it unveiled a significantly higher complication rate associated with VPS treatment.

In summary, our study’s findings align with those of previous research in the field. A Jesuyajolu et al. study [16] similarly observed no significant difference between VPS and ETV treatments, although they noted a slightly higher mortality risk of 0.05% in VPS compared to 0.01%. Sheik et al. study [17], which involved 100 patients treated with either VPS or ETV, suggested that VPS might carry a higher risk of long-term complications. In contrast, Idowu et al. study [18] found no statistically significant difference in complication rates between the two procedures. Notably, our study reported a higher success rate when compared to the existing literature. Specifically, we achieved a mean success rate ranging from 61.8 to 69.7% for ETV and 57.8 to 67.1% for VPS.

The paper’s fidelity to record complications in the context of the comparison between ETV and VPS for the treatment of paediatric hydrocephalus was based on the data sources and search methodology. We aimed to conduct a thorough systematic review and meta-analysis based on relevant and most inclusive databases such as PubMed, MEDLINE and the Cochrane Central Register of Controlled Trials in order to gather the most comprehensive set of studies. Further, we have included 8 RCTs and 1 prospective study which account for a strong study selection and methodologically strong approach to compare treatment outcomes. On top of that, our study clearly delineates the number of patients in the ETV and VPS groups and their respective complication rates which was later used in the statistical analysis. We have highlighted the *p*-values, combined success rates and median follow-up time for evaluating the accuracy of postoperative complication rates recording.

One of the implications of the study is to stratify a scoring tool that could predict the overall long-term success in high-, moderate-, and low-risk groups of paediatric hydrocephalus population. In turn, this could aid clinical decision-making when opting for either ETV or VPS treatment in the management of hydrocephalus. Durnford et al. study proposed a success validation score referred to as Endoscopic Third Ventriculostomy Success Score (ETVSS) in order to predict successful treatment for hydrocephalus based on the patient’s baseline characteristics [19]. The validation of this scoring tool was conducted on a population of 166 patients with hydrocephalus at a single neurosurgical centre in the UK. Our study recognises that by extending the validation tool of overall long-term success for both treatment methods of ETV and VPS for paediatric hydrocephalus patients, we would be able to stratify the clinical outcomes more realistically in management decision-making process.

The majority of RCTs in our systematic review and meta-analysis were representative of paediatric hydrocephalus population in African countries; hence, future research should address the need to obtain a greater representation of patient population from countries different than Africa.

### Limitations

One of the limitations of our meta-analysis was the small sample size included in 1 randomised controlled trial of Malheiros et al. study [3], which may potentially introduce bias against the generalisability of our data. We have set a minimum criterion to include 10 participants in our analysis; however, there is a scarcity of data in the paediatric population randomised controlled trials. Secondly, there was a paucity of research evidence in terms of various outcomes utilised. Our study showcased a heterogeneity of outcomes included and future research needs to address the need to mitigate the incomplete reporting of data. On top of that, the availability and quality of level I evidence included in this study were limited to 8 RCTs and 1 prospective study; hence, more randomised controlled trials are required to demonstrate the distinct advantage of either management approach in the paediatric population hydrocephalus. There was a limited number of RCTs with heterogenous methodological limitations in study protocols as aforementioned, potentially introducing reporting bias which may have affected the degree of our research robustness in our findings. Four studies reported incomplete reporting of outcomes, hence introducing “some concerns” and “high risk of bias” limitation of the overall quality and reliability of the evidence synthesised in this review and meta-analysis [1, 6, 9, 11]. Further, four studies were found to have “some concerns” in terms of the randomisation process which again may introduce reduced generalisability of our findings [1, 9, 12, 13].

## Conclusion

This is the first systematic review and meta-analysis focusing on the outcomes of ETV and VPS treatment as a management of choice for paediatric hydrocephalus. This meta-analysis demonstrates that endoscopic third ventriculostomy and ventriculoperitoneal shunt are both viable options for the surgical management with similar success rates reported. However, our study recognised that VPS results in a higher complication rate following the management of hydrocephalus in paediatric population. Although ETV has shown greater potential to treat paediatric hydrocephalus in comparison to the VPS treatment, more randomised controlled trials are needed to ascertain the effectiveness and the superiority of both treatments in the future research.

## Supplementary Information

Below is the link to the electronic supplementary material.

Supplementary file1 (DOCX 18 KB)

## Abbreviations

CPC: Choroid plexus cauterization
CSF: Cerebrospinal fluid
ETV: Endoscopic third ventriculostomy
PRISMA: Preferred Reporting Items for Systematic Reviews and Meta-Analyses
VPS: Ventriculoperitoneal shunt
