# Supplementary material for: Outcomes of endoscopic third ventriculostomy (ETV) and ventriculoperitoneal shunt (VPS) in the treatment of paediatric hydrocephalus: Systematic review and meta-analysis
Source: Childs Nerv Syst. 2023 Nov 27;40(4):1045–52. doi: 10.1007/s00381-023-06225-3 (PMC10972931; doi:10.1007/s00381-023-06225-3)
Supplement: Supplementary file 1 — Supplementary file1 (DOCX 18 KB) [file 381_2023_6225_MOESM1_ESM.docx]

**Appendix A**

Search protocol

**Section 1: Administrative Information**

**1. Title:** Outcomes of endoscopic third ventriculostomy (ETV) and ventriculoperitoneal shunt (VPS) in the treatment of paediatric hydrocephalus: systematic review and meta-analysis.

**2. Contributions:** KJM contributed to the conceptualisation, methodology, validation, formal analysis, investigation, data curation, writing – original draft, writing – review & editing, visualisation and project administration. SK contributed to the conceptualisation, methodology, validation, formal analysis, investigation, data curation, writing – original draft, writing – review & editing, visualisation and project administration. CK contributed to the conceptualisation, methodology, validation, resources, data curation, supervision and writing – review and editing.

**3. Amendments:** In the instance of the need for amendments to the protocol, the date of each amendment, a description of the change, and the rationale will be noted.

**4. Sponsor:** This review does not have any sponsoring agent.

**Section 2: Introduction**

**5**. **Objectives:** The objectives of this systematic review are as follows:

1. To evaluate the outcomes of endoscopic third ventriculostomy and ventriculoperitoneal shunt in the treatment of paediatric hydrocephalus.

2. To characterise the scope and the quality of current evidence and literature on endoscopic third ventriculostomy and ventriculoperitoneal shunt in the treatment of paediatric hydrocephalus.

**Section 3: Methods**

**6**. **Eligibility criteria:** Studies will be selected according to the criteria outlined below.

Study design

Inclusion criteria were as follows: randomized controlled trials, studies involving patients who had undergone an endoscopic third ventriculostomy or ventriculoperitoneal shunt as an intervention, studies involving paediatric population, studies involving human participants and including both genders, available full text articles published in English, patients who have hydrocephalus, studies found on the Ovid MEDLINE, Cochrane Register of Clinical Trials, Embase and Google Scholar databases and the timeline of RCTs was set between 2000 until present. The minimum sample size of the population was set to 10 participants for each RCT.

Participants

Paediatric population patients with hydrocephalus.

Interventions

Patients who have undergone endoscopic third ventriculostomy or ventriculoperitoneal shunt as an intervention.

Comparison

To compare the effectiveness of endoscopic third ventriculostomy and ventriculoperitoneal shunt in the management of hydrocephalus in paediatric population.

Outcomes

The primary outcomes of interest were treatment success. The secondary outcomes measured was complication rates.

Language

We included only articles reported in English.

**7**. **Information sources**: We searched PubMed, Cochrane Register of Controlled Trials and MEDLINE. The search strategy was developed by KJM and SK under the supervision of CK.

**8.Search Strategy:** PubMed, MEDLINE, and Cochrane Central Register of Controlled Trials databases were screened from April 3, 2023, until May 22, 2023. The search strategy was based on the utilisation of MeSH (Medical Subject Headings) term and each keyword search of each database was conducted based on the Boolean operators AND and OR. The searched keywords utilised in the article screening process included the following: “endoscopic third ventriculostomy”, “ventriculoperitoneal shunt”, “surgical management”, and “paediatric hydrocephalus”. The complete search protocol can be viewed in Appendix A. Thereafter, the data was uploaded to R programme for statistical analysis. We have reported our results based on descriptive statistics analysis and the forest plots obtained.

**9**. **Data Management:** Literature search results were uploaded to a data extraction

Spreadsheet in Excel, which allowed us to create a Preferred Reporting Items for Systematic Reviews and Meta-Analysis (PRISMA) flow diagram once the analysis was complete.

**10**. **Selection Process:** Authors KJM and SK independently evaluated the titles and abstracts of extracted articles to identify relevant studies meeting the inclusion criteria. At this stage, any duplicate articles were removed, and the selected articles were subjected to further review. Full articles were retrieved for all studies meeting the inclusion criteria, and the same two authors independently assessed the full text for applicability. In the case of any disagreements between the two authors, they discussed the matter to reach a consensus, and in certain instances, the senior supervisor CK was consulted to make the final decision. The reasons for excluding full-text articles were documented. Importantly, the reviewers were not blinded to the journal titles, institutions, or study authors during the review process.

**11**. **Outcomes and Prioritization**

Primary Outcome

Primary outcomes measured in the study was treatment success at 1 year post surgery.

Secondary outcomes

Secondary outcomes were complication rates.

**12**. **Criteria of Quantitative Synthesis (Data Synthesis):** A minimum of 5 research studies will be included for data synthesis as a minimum representative sample size. Primary and secondary outcomes will be extracted to Excel programme and tables will be created including descriptive statistics and aforementioned outcomes.

**13a**. **Proposed Additional Analyses (Data Synthesis):** None.

**13b**. **Type of Summary Planned if Quantitative Synthesis is Not Appropriate (Data**

**Synthesis):** If a quantitative analysis of data is deemed inappropriate, we will present

information in tables and text to summarize study characteristics and findings.
